# Pattern of item score change in Stroke Impairment Assessment Set in comprehensive inpatient rehabilitation wards

**DOI:** 10.20407/fmj.2019-010

**Published:** 2020-02-11

**Authors:** Kei Yagihashi, Shigeru Sonoda, Makoto Watanabe, Sayaka Okamoto, Yuko Okuyama, Hideto Okazaki

**Affiliations:** 1 Department of Rehabilitation Medicine II, Fujita Health University, School of Medicine, Tsu, Mie, Japan; 2 Department of Rehabilitation Medicine I, Fujita Health University, School of Medicine, Toyoake, Aichi, Japan; 3 Division of Rehabilitation, Fujita Health University Nanakuri Memorial Hospital, Tsu, Mie, Japan

**Keywords:** Cerebrovascular disorders, Impairment, Paralysis, Rehabilitation

## Abstract

**Objectives:**

Although numerous studies have examined activities of daily living (ADL) in stroke rehabilitation, there has been little focus on impairment, despite its close relationship to ADL. Therefore, we evaluated the change in impairment from admission to discharge of patients with stroke in comprehensive inpatient rehabilitation wards using the Stroke Impairment Assessment Set (SIAS).

**Methods:**

Data from 3279 patients with first stroke who were admitted to comprehensive inpatient rehabilitation wards between 2004 and 2016 were analyzed. A scattergram of the items showing the percentage of the highest score on admission and the percentage of patients whose score improved during hospitalization was plotted. The items of the SIAS were grouped by their location on the scattergram.

**Results:**

Three clusters could be discriminated on the scattergram. The upper right group, showed an improved score during hospitalization in combination with a high percentage of patients with the highest score on admission. This group consisted of the verticality, unaffected-side quadriceps, visuospatial, and pain items of the SIAS. The upper left group improved during hospitalization, but only contained a small percentage of patients with a high score on admission, and consisted of motor function items. The lower group was characterized by poor improvement during hospitalization and consisted of sensory, tone, range of motion, speech, and grip power items.

**Conclusions:**

Understanding the change in impairment during hospitalization using the three groups described above will facilitate design of a plan for stroke rehabilitation on admission.

## Introduction

Many studies have focused on activities of daily living (ADL) in stroke rehabilitation.^1–3^ The final ADL score is frequently predicted by the initial ADL status, age, degree of paralysis, visuospatial perception, presence of incontinence, or number of attacks.^4–7^ Compared with ADL, impairment is not predicted as frequently, and if it is, it is limited to several types of impairment, such as paralysis,^8,9^ aphasia,^10^ or visuospatial neglect.^11^ Further, the whole structure of impairment has been seldom studied.^12–14^

Therefore, we planned to evaluate the change in impairment using the Stroke Impairment Assessment Set (SIAS).^15,16^ The SIAS is a standardized instrument for stroke impairment, and includes items for motor function, muscle tone, sensation, range of motion, pain, trunk control, visuospatial perception, aphasia, and function of the unaffected side. Although several studies using the SIAS have mentioned the structure of impairment,^13,14^ only a few reports have examined how impairment changes over time.^17,18^

In the present study, we evaluated the change in the SIAS score from admission to discharge in patients with stroke in comprehensive inpatient rehabilitation wards.

## Methods

### Participants

A total of 4001 patients with first stroke who were admitted to our comprehensive inpatient rehabilitation wards between 2004 and 2016 were included in the current study. Patients who had a severe comorbidity, complications, or a falling accident, or those who showed deterioration of the Functional Independence Measure^19^ motor sub-score were excluded. Therefore, data from 3279 patients were analyzed. The full-time integrated treatment program consisted of rehabilitation therapy for 7 days a week. The ward with gyms and rooms for the patients was located face-to-face, and ensured that the patients were active during the daytime (e.g., moving around in their wheelchair, interacting with other patients or their families, or engaging in other activities).^20^ Therefore, systematic and homogeneous rehabilitation was provided to all patients.

### Ethics

This study was approved by the ethics committee of Fujita Health University (approval no. HM19-006).

### Methods

Impairment was evaluated on admission and at discharge using the SIAS. The SIAS is primarily based on single-task assessment of various functions, where performance is rated on scales of 0–5 or 0–3, with higher scores indicating less impairment. The SIAS consists of items examining motor function, muscle tone, sensory function, range of motion, pain, trunk balance, visuospatial perception, aphasia, and function of the unaffected side (Table 1). The percentage of patients for each score of each item of the SIAS on admission and at discharge was counted.

SIAS item scores at admission and discharge were cross-tabulated. The number of patients with the highest possible score (i.e., with the least impairment on admission) was counted and the percentage against all patients was calculated. Further, the number and percentage of patients whose score improved during hospitalization were calculated. Patients whose score was highest on admission were excluded in advance. A scattergram of the items showing the percentage of patients with the highest score on admission and the percentage of patients whose score improved during hospitalization was plotted. The SIAS items were visually grouped according to their location on this scattergram.

## Results

**Figure 1:**
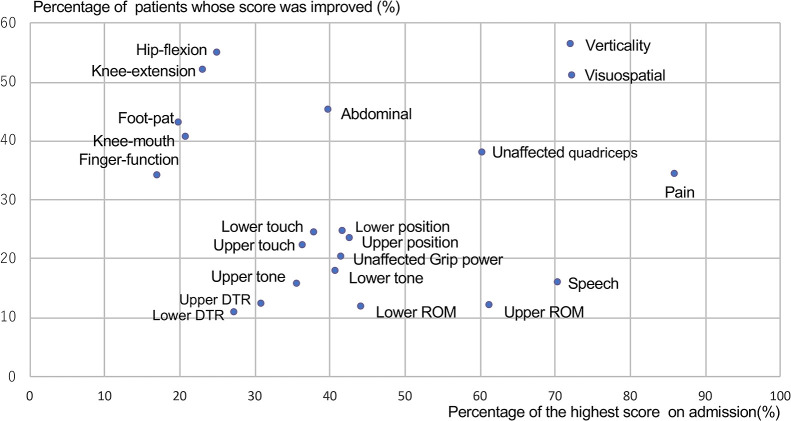
Scattergram of the Stroke Impairment Assessment Set items in patients with stroke. The upper right group consists of the following items: verticality, unaffected-side quadriceps, visuospatial, and pain. The upper left group consists of the following five motor function items: hip flexion, knee extension, foot pad, knee mouth, and finger function. The lower group consists of the following items, where only a small percentage of patients showed an improved score during hospitalization: sensory, tone, range of motion, speech, and grip power. DTR, deep tendon reflex; ROM, range of motion.

Table 2 shows the demographic data of the patient cohort. The average time from onset to admission and the average duration of hospitalization were 37 days and 56 days, respectively. Table 3 shows the percentage of patients for each score of each item of the SIAS on admission and at discharge. Figure 1 shows a scattergram of the SIAS items showing the percentage of patients with the highest score on admission and the percentage of patients with an improved score during hospitalization. Three clusters (i.e., upper right, upper left, and lower groups) were able to be discriminated. A high percentage of patients who improved during hospitalization, in combination with a high percentage of patients with the highest score on admission, were characteristic of the upper right group. This group included verticality, unaffected-side quadriceps, visuospatial, and pain items. The upper left group, who showed improvement during hospitalization, but contained a small percentage of patients with the highest score on admission, consisted of motor function items. The abdominal manual muscle testing (MMT) item was located midway between the upper right and upper left groups. The lower group, which was characterized by poor improvement during hospitalization, consisted of sensory (touch and position), tone, and range of motion items for the upper and lower extremities, and speech and grip power items.

## Discussion

The SIAS used in the current study examines impairments that are frequently encountered during stroke rehabilitation. All SIAS tests can be performed in a seated position.^15,16^ Therefore, the SIAS can be easily used in the clinical setting and be applied to determine a rehabilitation plan, making it an important element of treatment.

The current study showed that impairment items in the SIAS were able to be divided into three groups in patients with stroke. The factors used to classify the items were related to the change in impairment during hospitalization. Knowing the possibility of improvement in impairment would facilitate deciding which items should be addressed predominantly in the early phase of rehabilitation. The characteristics of the three groups are discussed below.

### Upper right group

The upper right group consisted of verticality, unaffected-side quadriceps, visuospatial, and pain items. The unaffected-side quadriceps items are related to the immobilization state. Because early-phase rehabilitation is commonly introduced and unnecessary immobilization tends to be avoided in Japan, the frequency of immobilized patients is low.^21^ As a result, the percentage of patients with the highest score on admission for these items was high because immobilization decreases the scores of these items. Even in the presence of immobilization, a high improvement rate is expected because most of the negative effects of immobilization that are present for approximately 1 month after stroke can be improved by exercise.^22^ Furthermore, the full-time integrated treatment program, which we used in the current study, is based on motor learning theory and emphasizes gait exercise.^20^ Therefore, this program promotes improvement of these items. The reason for the presence of verticality in this group is bilateral innervation of trunk muscles,^23^ which reduces impairment and facilitates better improvement following exercise.

The symptoms of visuospatial neglect occur less frequently in patients with brain lesions in the left hemisphere compared with those with right hemisphere lesions. Therefore, the percentage of patients with a score of 3 in the visuospatial item was high because of the existence of patients with left brain lesions. Ringman et al.^11^ reported that the occurrence of hemispatial neglect at 3 months decreased from 43% to 17% in patients with a right hemisphere lesion, while it decreased from 17% to 5% in those with a left hemisphere lesion. Pain that is not related to stroke, such as that derived from osteoarthritis, is not included in the pain score. The percentage of patients with a score of 3 in this item was high in our study. A reduction in shoulder pain after stroke was frequently observed and this phenomenon contributed to a good improvement ratio for this item in the current study.

### Upper left group

All five items included in the upper left group were motor paralysis items. The high occurrence of hemiplegia can be attributed to it being a major target of rehabilitation, therefore prompting the tendency for patients with hemiplegia to be transferred to a rehabilitation hospital. The hospitalization period of the current cohort was an average of 37 days since onset on admission and 100 days since onset at discharge. This timing coincides with the improvement period of motor paralysis. Jorgensen et al. reported that arm function recovery was completed within 12.5 weeks from stroke onset in 95% of patients.^24^ Patients with mild and severe upper extremity paresis achieve the best possible function within 3–6 weeks and 6–11 weeks, respectively.^25^ These findings correlate with the high number of patients whose hemiplegia improved in the current study. Compared with the other three motor paralysis items, hip flexion and knee extension items showed a relatively high rate of improvement in our study. These items evaluate the proximal function of the lower limbs. The large number of gait exercise sessions provided to patients would have mobilized the proximal muscles of the lower extremities, inducing an improvement in motor paralysis.^18,26^

The abdominal MMT item was located midway between the upper right and upper left groups. This could be attributed to bilateral innervation of trunk muscles^27^ because the factors of contralateral paralysis and disuse affect the abdominal MMT item.

### Lower group

The lower group consisted of items for which only a small percentage of patients had an improved score. These items were as follows: sensory, tone, range of motion, speech, and grip power. The proportions of scores for each item in this group on admission and at discharge were not different (Table 3), and a specific pattern for improvement or no improvement was not found in this study. Further detailed analysis, such as performing cross table analysis in each item, is required to discriminate patients who improve or do not improve. However, this will require a much higher number of patients for analysis. Sensory disturbance was less improved after treatment compared with rehabilitation of motor impairment or ADL because there are few methods to improve sensory disturbance. Urban et al.^28^ assessed 211 patients of whom 42.6% developed spasticity at approximately 6 months after stroke. The natural course of spasticity varies, with it increasing in some cases and decreasing in others.^29^ Accentuated tone and restriction of the range of motion of joints are sometimes related to each other,^30^ lowering the improvement rate of these items.

Sarno^31^ reported that fluent aphasia improved during the first 6 months after stroke, while non-fluent aphasia required more time for recovery. Although total aphasia did not improve much, there was some improvement after 6 months. Because the definition of the speech item in the SIAS is subjective and vague (i.e., mild, moderate, and severe), certain improvement of aphasia during hospitalization might not be reflected in the SIAS score of some patients.

Domen et al.^32^ reported that unaffected-side grip power from admission to discharge during stroke inpatient rehabilitation increased by approximately 2 kg. Therefore, there is a possibility that a change in unaffected-side grip power may be undetected by the SIAS because the grip power score in the SIAS is separated by 3, 10, and 25 kg.^15,16^

### Study limitations

Although the scores of the SIAS items are unified into scales of 0–5 and 0–3 for motor paralysis and other items, respectively, the individual definitions of each score are not integrally controlled. Therefore, a change in the score of each item may not be weighted equally. Finally, because the current study was observational, we could not discriminate whether the improvements were due to exercise or the natural course of recovery. A future interventional study is required to clarify this issue.

## Conclusion

The impairment items in the SIAS can be divided into three groups in patients with stroke. Understanding the change in impairment during hospitalization will facilitate planning stroke rehabilitation on admission.

## Figures and Tables

**Table1:** Items and ratings of the Stroke Impairment Assessment Set

		Upper extremity	Lower extremity
Motor function	Proximal	0–5 (Knee-mouth)	0–5 (Hip-flexion)
			0–5 (Knee-extension)
	Distal	0–5 (Finger-function)	0–5 (Foot-pat)
Tone	DTR’s	0–3	0–3
	Muscle tone	0–3	0–3
Sensory function	Touch	0–3	0–3
	Position	0–3	0–3
ROM		0–3 (Upper ROM)	0–3 (Lower ROM)
Pain		0–3	
Trunk balance	Abdominal	0–3	
	Verticality	0–3	
Visuospatial		0–3	
Speech		0–3	
Unaffected side		0–3 (Grip)	0–3 (Quadriceps)

A larger score indicates a smaller impairment. Abbreviations. DTR: deep tendon reflex, ROM: range of motion, and MMT: manual muscle testing. Scale ranges are shown as 0–5 or 0–3.

**Table2:** Demographic data of the subjects in this study

Average age (years; mean±sd)	65±13
Sex (male:female)	1972/1307
Disease (cerebral infarction/intracerebral hemorrhage/subarachnoid hemorrhage)	1616/1414/249
Length of stay (days; mean±sd)	63±39
Average days from onset to admission (days; mean±sd)	37±38

sd: standard deviation

**Table3:** The percentage of patients of each score of each item of the SIAS on admission and at discharge

		0	1	2	3	4	5
Knee-mouth	Admission	19	16	11	12	21	21
	Discharge	10	14	12	12	25	26
Finger-function	Admission	28	19	6	7	22	17
	Discharge	19	21	7	7	24	22
Hip-flexion	Admission	14	12	14	10	25	25
	Discharge	5	8	13	11	29	34
Knee-extension	Admission	17	9	14	12	25	23
	Discharge	7	7	13	13	28	32
Foot-pat	Admission	26	9	9	10	26	20
	Discharge	16	8	10	10	28	28
Upper DTR	Admission	3	31	39	27		
	Discharge	3	29	40	28		
Lower DTR	Admission	3	30	39	28		
	Discharge	3	27	41	29		
Upper M tone	Admission	1	30	33	36		
	Discharge	1	25	36	37		
Lower M tone	Admission	1	25	33	41		
	Discharge	2	22	34	42		
Upper touch	Admission	9	23	31	37		
	Discharge	5	21	33	41		
Lower touch	Admission	9	23	30	38		
	Discharge	4	20	33	43		
Upper position	Admission	14	19	24	43		
	Discharge	9	19	25	47		
Lower position	Admission	13	20	25	42		
	Discharge	8	19	27	46		
Upper ROM	Admission	3	16	20	61		
	Discharge	3	16	21	60		
Lower ROM	Admission	2	10	44	44		
	Discharge	1	9	45	45		
Quadriceps	Admission	1	9	30	60		
	Discharge	1	5	23	71		
Grip	Admission	4	12	43	41		
	Discharge	3	8	42	47		
Pain	Admission	1	2	10	86		
	Discharge	1	2	9	88		
Verticality	Admission	6	9	13	72		
	Discharge	3	4	10	83		
Abdominal	Admission	13	18	29	40		
	Discharge	6	11	30	53		
Speech	Admission	4	12	13	70		
	Discharge	3	12	12	73		
